# Efficacy of coenzyme Q10 supplementation on glucose metabolism, lipid profiles, and biomarkers of inflammation in women with polycystic ovary syndrome: A protocol for a systematic review and meta-analysis

**DOI:** 10.1097/MD.0000000000023130

**Published:** 2020-11-13

**Authors:** Min Liu, Hongqiu Zhu, Xiaodan Hu, Ying Zhu, Haiyan Chen

**Affiliations:** a Hospital of Chengdu University of Traditional Chinese Medicine; b Department of Gynaecology, School of Medical and Life Sciences, Chengdu University of Traditional Chinese Medicine/Reproductive & Women-Children Hospital of Chengdu University of Traditional Chinese, Chengdu City, Sichuan Province, China.

**Keywords:** coenzyme Q10, glucose metabolism, inflammation, lipid profiles, polycystic ovary syndrome, protocol

## Abstract

**Background:**

Polycystic ovary syndrome (PCOS) is one of the common gynecological endocrine system diseases. It is characterized by excessive androgen, rare or anovulation, and polycystic ovary morphology. Coenzyme Q10 (CoQ10) is a fat-soluble natural vitamin, which has a continuous oxidation-reduction cycle and is an effective antioxidant that can protect ovaries from oxidative damage. This study aims to systematically summarize and analyze the scientific literatures on glucose metabolism index, lipid profiles, inflammatory factor, and sex hormone level of PCOS patients treated with CoQ10 to provide a reference basis for clinical treatment.

**Methods:**

We will retrieve the following electronic databases from the built-in until March 2021: Cochrane Library, PubMed, EMBASE, Web of Science, China National Knowledge Infrastructure (CNKI), Chinese Biomedical Literature Database (CBM), Clinical Trials. gov, Chinese Scientific Journal Database (VIP), and Wang-fang database. Two reviewers will independently scan the articles searched, de-duplication, filtering, quality assessment. Differences will be resolved by discussion between the 2 reviewers or by a third reviewers. All analyses were systematic to evaluate interventions based on the Cochrane handbook. Meta-analysis and/or subgroup analysis will be performed on the basis of the included studies.

**Discussion:**

This review will be to investigate the efficacy of CoQ10 supplementation on glucose metabolism, lipid profiles, and biomarkers of inflammation in women with PCOS and provide a high-quality synthesis to assess whether CoQ10 is an effective and safe intervention for PCOS. The results of the analysis will be published in a scientific journal after peer-review.

**Systematic review registration:**

INPLASY 2020100013.

## 1 Introduction

Polycystic ovary syndrome (PCOS) is the most common endocrine disorder in women of reproductive age.^[1]^ Insulin resistance, increased free fatty acids (FFAs), and increased obesity are the key factors causing metabolic dysfunction in PCOS women.^[2]^ More than 10% of women have PCOS, which is characterized by ovulatory dysfunction, high clinical levels of biochemical androgen, and polycystic ovaries. Metabolic sequelae associated with PCOS include insulin resistance (IR), type 2 diabetes (T2DM), obesity, and increased cardiometabolic risk.^[3,4]^

Coenzyme Q10 (CoQ10) is a fat-soluble natural vitamin, which has a continuous oxidation-reduction cycle and is an effective antioxidant^[5]^ that can protect ovaries from oxidative damage.^[6]^ Studies have shown that dietary supplementation of CoQ10 can improve the metabolic and endocrine indexes^[7]^ of PCOS patients, as well as insulin resistance^[8]^ and endothelial cell function.^[9]^ Rahmani et al^[7]^ found that after 12 weeks of oral administration of 100 mg CoQ10 per day, the serum fasting plasma glucose (FPG), fasting insulin (FINS), and homeostasis model assessment of insulin resistance (HOMA-IR) levels of PCOS patients were significantly reduced. Similarly, a meta-analysis^[8]^ reported that CoQ10 could reduce FPG and HbA1c. Studies have shown that CoQ10 can improve glucose metabolism indicators in patients with different pathological conditions, such as nonalcoholic fatty liver disease,^[10]^ diabetes mellitus, and metabolic syndrome.^[11]^ Schroeder et al^[12]^ suggested that interleukin-1 (IL-1) inhibits the high glycemic release of insulin, while CoQ10 blocks the inhibition of IL-1. Low dose of CoQ10 can promote the differentiation of islet progenitor cells. CoQ10 is an effective antioxidant, which has been proven to prevent lipid and protein peroxidation and scavenging free radicals in the body and cells.^[13]^ If CoQ10 is deficient, the above process will be abnormal, free radical accumulation, and oxidative stress damage will be aggravated. CoQ10 can regulate the amount and function of inflammatory factors, insulin receptors, adiponectin receptors, glucose transporters, tyrosine kinases, phosphatidylinositol kinases, and oxygen free radicals, so as to regulate glycolipid metabolism and improve insulin resistance.

This study aims to systematically summarize and analyze the scientific literatures on glucose metabolism index, inflammatory factor, and sex hormone level of PCOS patients treated with CoQ10, so as to provide a reference basis for clinical treatment.

## 2 Methods

### 2.1 Study registration

The protocol has been registered in the International platform of registered systematic review and meta-analysis protocols (INPLASY) as number INPLASY2020100013, including prespecified analysis plan, the full text analysis program can be obtained at inplasy.com (https://doi.org/10.37766/inplasy2020.10.0013). It will follow the recommendations of the Cochrane handbook for systematic reviews of interventions^[14]^ and will be constructed based on the Preferred Reporting Items for Systematic Review and Meta-Analysis Protocols (PRISMA-P).^[15]^

### 2.2 Search strategy

We will retrieve the following electronic databases from the built-in until March 2021: Cochrane Library, PubMed, EMBASE, and Web of Science, China National Knowledge Infrastructure (CNKI), Chinese Biomedical Literature Database (CBM), Clinical Trials. gov, Chinese Scientific Journal Database (VIP), and Wang-fang database. We adopt the combination of heading terms and free words as search strategy, which will be decided by all the reviewers. We will apply a search method combining MeSH terms and free words. Search terms will be as follows: Polycystic ovary syndrome, PCOS, coenzyme Q10, CoQ10, randomized controlled trials (RCTs), etc. The search strategy on PubMed is provided in Table 1. Meanwhile, we will retrieve other resources to complete the deficiencies of the electronic databases, primarily searching for the grey literature on the corresponding website. The search results will be limited to human studies, and all included studies will be RCTs with no language limitations. If necessary, we will contact the corresponding author of the relevant study by e-mail or telephone to obtain sufficient information.

**Table 1:** Search strategy in PubMed database.

No.	Search items
1	Coenzyme Q10. [mesh]
2	CoQ10. [mesh]
3	1 or 2
4	Polycystic Ovary Syndrome. [mesh]
5	Polycystic Ovarian Syndrome. [mesh]
6	PCOS. [mesh]
7	4 or 5 or 6
8	Randomized controlled trial. [pt]
9	Controlled clinical trial. [pt]
10	Clinical trials as topic [mesh: noexp]
11	Randomized [ti, ab]
12	Randomly. [ti, ab]
13	Placebo. [ti, ab]
14	Trial. [ti, ab]
15	8 OR 9 OR 10 OR 11 OR 12 OR 13 OR 14
16	3 AND 7 AND 15

### 2.3 Inclusion criteria

#### 2.3.1 Type of study

The included studies will be limited to human studies with RCTs, and the use of random or blind methods and published language were not restricted. If an experiment does not explain the random method or does not explain the randomization, the study will be considered to be at high risk for random sequence generation.

#### 2.3.2 Types of participant

Adult women diagnosed with PCOS according to Rotterdam criteria in 2003. Adolescents (under 18) and postmenopausal women (over 50) will be excluded from the review.

#### 2.3.3 Type of intervention

Intervention strategies include CoQ10 or CoQ10 combined with traditional Chinese medicine (TCM) or western medicine or lifestyle interventions such as diet and exercise. CoQ10 can be used at any dose, frequency, and duration.

#### 2.3.4 Type of comparators

The control included blank, placebo, TCM treatment, western medicine treatment, or lifestyle intervention such as diet and exercise.

#### 2.3.5 Types of outcomes

Primary outcomes consist of menstrual cycle regulation, body mass index (BMI), HOMA-IR, luteinizing hormone (LH), follicle-stimulating hormone (FSH), testosterone (T), estradiol (E2), progesterone (P), serum sex hormone binding globulin (SHBG), dehydroepiandrosterone sulphate (DHEAS), and free androgen index (FAI). Secondary outcomes were Ferriman-Gallway score, acne score, waist to hip ratio (WHR), total cholesterol (TC), triglyceride (TG), low-density lipoprotein (LDL), high-density lipoprotein (HDL), fasting plasma glucose (FPS), FINS, insulin sensitivity index (ISI), interleukin-1 (IL-1), interleukin-8, tumor necrosis factor alpha (TNF-α), adverse reactions, etc.

### 2.4 Exclusion criteria

Exclusion criteria included adolescents (under 18 years of age), postmenopausal women (over 50 years of age), pregnant and lactation women; patients who had taken drugs affecting hormone levels, such as antioxidants, ovulation inducers, or oral contraceptives, within 3 months before the experiment; other causes of menstrual disorders and androgen overload, such as congenital adrenal hyperplasia, androgen secreting tumors, Cushing syndrome, etc.

### 2.5 Data collection

#### 2.5.1 Data management

Endnote X9.1 software was used to manage the retrieved literature and delete duplicate literature. Two researchers trained in methodology independently selected the relevant literature. If there is a difference, a third investigator will decide. We will then extract the data into a Microsoft Excel spreadsheet. The extracted data will be analyzed and synthesized by the Review Manager software (RevMan V.5.3).

#### 2.5.2 Data extraction

**Figure 1:**
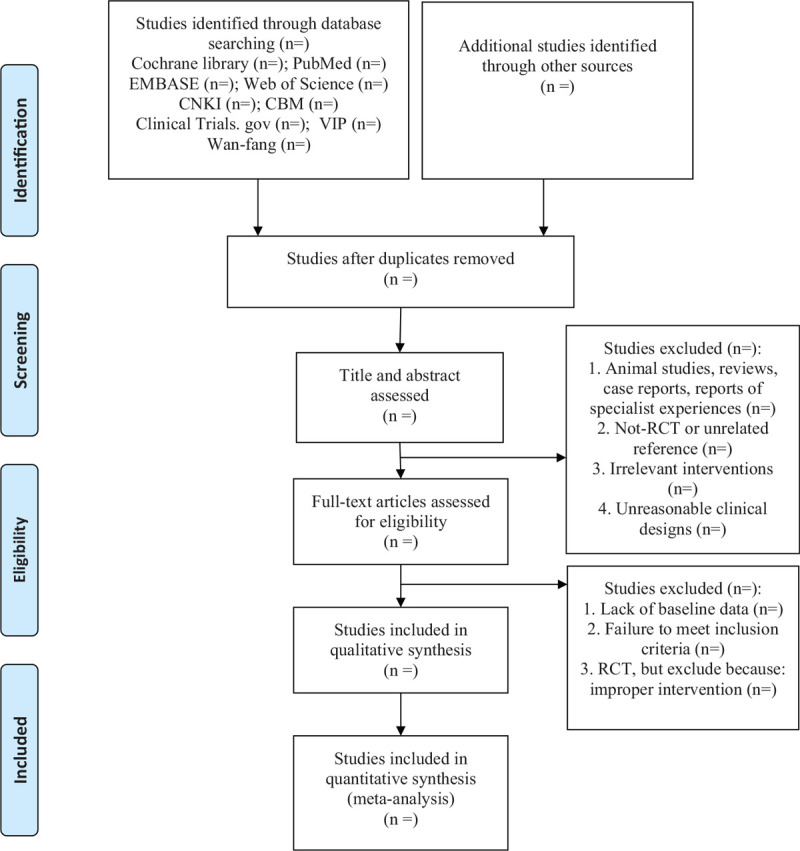
PRISMA flow diagram of the study process.

Two independent reviewers will draw on the PRISMA statement's recommendations for data extraction; PRISMA flowchart will be used to show the citation selection process of the study (Fig. 1). Differences between the 2 reviewers will be resolved by a third reviewer to reach a consensus. All citations will be imported into the reference management software Endnote X9.1, and repeated citations will be deleted automatically. We will assess the eligibility of the remaining studies step by step through examining the titles, abstracts, and full texts. The data extracted will be as follows:

(1) The basic information of the study: authors, location, language, title, and publication time.
(2) Inclusion and exclusion criteria.
(3) The baseline of the study: the sample size, average age, race.
(4) Intervention and control: CoQ10 and/or other drug, dosage, frequency, route of administration, duration of treatment, etc.
(5) Methodological information: description for randomization, sequence concealment, blinding, incomplete outcome data, selective reporting, and other potential risk of bias.
(6) outcome measures.

If necessary, the corresponding author of the selected publication will be contacted for missing data and more information.

#### 2.5.3 Risk of bias assessment

Two authors will independently evaluate the risk of bias using the approach recommended by Cochrane Handbook for Systematic Reviews of Interventions.^[14]^ The following 6 risk of bias domains will be assessed: random sequence generation (selection bias); allocation concealment (selection bias); blinding of participants, personnel (performance bias); blinding of outcome assessment (detection bias); incomplete outcome data (attrition bias); selective outcome reporting (reporting bias); and other bias. It visually explains the quality of articles, using greens, reds, and yellows, as well as “+,” “-,” “?” The symbol is on behalf of “low risk bias,” “high risk bias,” and “unclear” to assess the risk of included studies. A funnel plot will be drawn to assess the reporting bias. Asymmetric funnel plot shows high risk of report bias, while symmetric funnel plot shows low risk of report bias. After the assessment, reconfirmation will be conducted. If there are differences between the 2 authors, the results will be unified after mutual discussion or judged by the third author.

#### 2.5.4 Dealing with missing data

If the data are not reported, we may contact the corresponding author via email or phone to request missing data, especially those necessary to complete the meta-analysis.

### 2.6 Statistical analysis

#### 2.6.1 Data synthesis

All analyses will be conducted with Review Manager V.5.3 software. For continuous data, if there is no heterogeneity, we will use mean difference (MD) or standard MD (SMD) to measure the treatment effect of 95% CI. For dichotomous data, we will use a 95% CI risk ratio (RR) for analysis. When statistical heterogeneity is low, the fixed effect model will be used to merge data. However, when *P* < .1 or *I*^*2*^ > 50%, the random effect model will be used to provide a more conservative estimate of the effects. When *I*^*2*^ > 75%, we will look for possible causes from clinical and methodological perspectives, and provide a descriptive analysis or subgroup analysis. If a meta-analysis is not possible, we will provide a narrative summary of the results from individual studies.

#### 2.6.2 Subgroup analysis

If there is significant heterogeneity in study results, we will conduct subgroup analysis according to different reasons. The heterogeneity was mainly manifested in race, age, gender, coenzyme Q10 dose and course of treatment, etc.

#### 2.6.3 Sensitivity analysis

Sensitivity analysis was used to evaluate the robustness of the main efficacy indicators. The method is to eliminate the low-quality studies one by one, merge the data, and evaluate the impact of sample size, statistical methods, study quality, and missing data on the analysis results.

#### 2.6.4 Assessment of reporting bias

In this analysis, reporting bias will be explored by constructing funnel plots and performing Egger test, if there are at least 10 trials included.

#### 2.6.5 Quality of evidence

On the basis of the recommendations from the Cochrane Handbook for Systematic Reviews of Interventions, the Grading of Recommendations Assessment, Development and Evaluation (GRADE) system will be used to assess the quality of the evidence for all outcomes.^[16]^ The system takes into account risk of bias, consistency, directness of evidence, accuracy of effect estimation, and publication bias within the study. In order to achieve transparency, the overall quality and strength of the evidence will be accurately classified into 4 levels: high, medium, low, and very low (Table 2).

**Table 2:** Quality of evidence and definitions.

High quality	Further research is very unlikely to change the confidence in the estimate of effects
Moderate quality	Further research is likely to have an important impact on the confidence in the estimate of effect and may change the estimate
Low quality	Further research is very likely to have an important impact on the confidence in the effect and is likely to change the estimate
Very low quality	Any estimate of the effect is very uncertain

#### 2.6.6 Ethics and dissemination

Ethical considerations are unnecessary. The results will be published in a peer-reviewed journal based on PRISMA guidelines.^[17,18]^ This systematic review aims to explore the role of CoQ10 in improving glucose metabolism, lipid metabolism, and inflammation in PCOS women.

## 3 Discussion

Patients with PCOS often have abnormal glucose tolerance or type 2 diabetes.^[19,20]^ This study found that the use of CoQ10 significantly reduced serum FPG and HOMA-IR. CoQ10 can effectively improve glucose and lipid metabolism, inflammation, and sex hormone levels, but there is still a lack of large sample size, multicenter, high-quality, double-blind, RCT to verify. Therefore, this study aims to evaluate the efficacy and safety of CoQ10 in the treatment of PCOS. The protocol may have some limitations, but if there are any significant revisions, we will present the date of each amendment with a description of the change and the corresponding rationale in INPLASY website. The results of the systematic review will be published in peer-reviewed open access opinion journals to enable relevant scientific and clinical staff to obtain these results and provide convincing evidence for clinicians to make decisions.

## Author contributions

**Conceptualization:** Min Liu, Hongqiu Zhu.

**Data curation:** Xiaodan Hu, Haiyan Chen.

**Investigation:** Min Liu, Hongqiu Zhu, Ying Zhu.

**Methodology:** Min Liu, Hongqiu Zhu.

**Project administration:** Min Liu, Hongqiu Zhu, Xiaodan Hu.

**Writing – original draft:** Min Liu.

**Writing – review & editing:** Min Liu, Hongqiu Zhu.

## References

[1] Zhao X Jiang Y Xi H Exploration of the relationship between gut microbiota and polycystic ovary syndrome (PCOS): a review. Geburtshilfe Frauenheilkd 2020;80:161–71. 32109968 10.1055/a-1081-2036 PMC7035130

[2] Siemienowicz KJ Coukan F Franks S Aberrant subcutaneous adipogenesis precedes adult metabolic dysfunction in an ovine model of polycystic ovary syndrome (PCOS). Mol Cell Endocrinol 2020;519:111042. 33010309 10.1016/j.mce.2020.111042

[3] Abdalla M Deshmukh H Atkin SL miRNAs as a novel clinical biomarker and therapeutic targets in polycystic ovary syndrome (PCOS): a review. Life Sci 2020;259:118174. 32745529 10.1016/j.lfs.2020.118174

[4] Wekker V van Dammen L Koning A Long-term cardiometabolic disease risk in women with PCOS: a systematic review and meta-analysis. Hum Reprod Update 2020;26:942–60. 32995872 10.1093/humupd/dmaa029 PMC7600286

[5] Bentinger M Brismar K Dallner G The antioxidant role of coenzyme Q. Mitochondrion 2007;7:S41–50. 17482888 10.1016/j.mito.2007.02.006

[6] Ozcan P Ficicioglu C Kizilkale O Can Coenzyme Q10 supplementation protect the ovarian reserve against oxidative damage? J Assist Reprod Genet 2016;33:1223–30. 27255570 10.1007/s10815-016-0751-z PMC5010809

[7] Rahmani E Jamilian M Samimi M The effects of coenzyme Q10 supplementation on gene expression related to insulin, lipid and inflammation in patients with polycystic ovary syndrome. Gynecol Endocrinol 2018;34:217–22. 28949260 10.1080/09513590.2017.1381680

[8] Stojanovic M Radenkovic M A meta-analysis of randomized and placebo-controlled clinical trials suggests that coenzyme Q10 at low dose improves glucose and HbA1c levels. Nutr Res 2017;38:1–2. 28381349 10.1016/j.nutres.2016.12.001

[9] Yang YK Wang LP Chen L Coenzyme Q10 treatment of cardiovascular disorders of ageing including heart failure, hypertension and endothelial dysfunction. Clin Chim Acta 2015;450:83–9. 26254995 10.1016/j.cca.2015.08.002

[10] Farhangi MA Alipour B Jafarvand E Oral coenzyme Q10 supplementation in patients with nonalcoholic fatty liver disease: effects on serum vaspin, chemerin, pentraxin 3, insulin resistance and oxidative stress. Arch Med Res 2014;45:589–95. 25450583 10.1016/j.arcmed.2014.11.001

[11] Raygan F Rezavandi Z Dadkhah Tehrani S The effects of coenzyme Q10 administration on glucose homeostasis parameters, lipid profiles, biomarkers of inflammation and oxidative stress in patients with metabolic syndrome. Eur J Nutr 2016;55:2357–64. 26385228 10.1007/s00394-015-1042-7

[12] Schroeder MM Belloto RJ Jr Hudson RA Effects of antioxidants coenzyme Q10 and lipoic acid on interleukin-1 beta-mediated inhibition of glucose-stimulated insulin release from cultured mouse pancreatic islets. Immunopharmacol Immunotoxicol 2005;27:109–22. 15803864 10.1081/iph-51755

[13] Mohammadi-Bardbori A Najibi A Amirzadegan N Coenzyme Q10 remarkably improves the bio-energetic function of rat liver mitochondria treated with statins. Eur J Pharmacol 2015;762:270–4. 26007644 10.1016/j.ejphar.2015.05.041

[14] Cumpston M Li T Page MJ Updated guidance for trusted systematic reviews: a new edition of the Cochrane Handbook for Systematic Reviews of Interventions. Cochrane Database Syst Rev 2019;10:ED000142. 31643080 10.1002/14651858.ED000142 PMC10284251

[15] Shamseer L Moher D Clarke M Preferred reporting items for systematic review and meta-analysis protocols (PRISMA-P) 2015: elaboration and explanation. BMJ 2015;350:g7647. 25555855 10.1136/bmj.g7647

[16] Balshem H Helfand M Schunemann HJ GRADE guidelines: 3. Rating the quality of evidence. J Clin Epidemiol 2011;64:401–6. 21208779 10.1016/j.jclinepi.2010.07.015

[17] Knobloch K Yoon U Vogt PM Preferred reporting items for systematic reviews and meta-analyses (PRISMA) statement and publication bias. J Craniomaxillofac Surg 2011;39:91–2. 21145753 10.1016/j.jcms.2010.11.001

[18] Moher D Liberati A Tetzlaff J Preferred reporting items for systematic reviews and meta-analyses: the PRISMA statement. Int J Surg 2010;8:336–41. 20171303 10.1016/j.ijsu.2010.02.007

[19] Codner E Type 1 diabetes, obesity and PCOS: is type 1 stepping into the shoes of type 2 diabetes? Clin Endocrinol (Oxf) 2020. 10.1111/cen.14319 32945565

[20] Lewandowski KC Plusajska J Horzelski W Prevalence of dyslipidaemia and pre-diabetes among women with polycystic ovary syndrome (PCOS): do we overestimate cardiovascular risk? Horm Metab Res 2019;51:539–45. 31075796 10.1055/a-0896-4144

